# Retraction Note: MCM3AP-AS1/miR-876-5p/WNT5A axis regulates the proliferation of prostate cancer cells

**DOI:** 10.1186/s12935-024-03381-7

**Published:** 2024-05-29

**Authors:** Jie Wu, Yalin Lv, Yujun Li, Yanxia Jiang, Lili Wang, Xiangyan Zhang, Mengqi Sun, Yuwei Zou, Jin Xu, Li Zhang

**Affiliations:** 1 Department of Pathology, The Affiliated Hospital of Qingdao University, Jiangsu Road, South District, Qingdao, 266003 Shandong China; 2 Department of Dermatology, The Affiliated Hospital of Qingdao University, Qingdao, 266003 China

**Retraction Note: Cancer Cell International (2020) 20:307**

10.1186/s12935-020-01365-x

The Editors have retracted this article on the corresponding author’s request. After the publication of this article, concerns have been raised regarding image overlap between Fig. 5e (si-con, KYSER450) and Fig. 2e (miR-Control, PC3) of [1]. The corresponding author stated that the fluorescence and flow cytometry experiments were completed by a third party and they don’t have any raw data as well as evidence for ethics approval.

Therefore, The Editors no longer have confidence in the results and conclusions presented in this article.

Authors, Li Zhang and Jie Wu agree to this retraction. Authors, Yalin Lv, Yujun Li, Yanxia Jiang, Lili Wang, Xiangyan Zhang, Menggi Sun, Yuwei Zou, and Jin Xu have not responded to any correspondence from the editor/publisher about this retraction.
